# Comparative genomics of an Antarctic sea-ice diatom (*Nitzschia* sp.) provides insights into potential polar adaptation

**DOI:** 10.3389/fmicb.2026.1755917

**Published:** 2026-04-23

**Authors:** Chuan Zhai, Fraser Kennedy, Meiaoxue Han, Hao Yu, Jianhua Sun, Yantao Liang, Min Wang, Andrew McMinn

**Affiliations:** 1 Institute for Marine and Antarctic Studies, University of Tasmania, Hobart, TAS, Australia; 2 The Australian Centre for Excellence in Antarctic Science, University of Tasmania, Hobart, TAS, Australia; 3 Key Lab of Polar Oceanography and Global Ocean Change, College of Marine Life Sciences, Institute of Evolution and Marine Biodiversity, Frontiers Science Center for Deep Ocean Multispheres and Earth System, Ocean University of China, Qingdao, China; 4 UMT-OUC Joint Centre for Marine Studies, Qingdao, China; 5 Haide College, Ocean University of China, Qingdao, China

**Keywords:** adaptation, diatom, EVE, genomic, polar, sea ice, TE

## Abstract

Sea-ice diatoms are key primary producers in Antarctic sea-ice ecosystems and the Southern Ocean. However, the genomic features that characterize these organisms remain incompletely understood. Here, we present a high-quality draft genome assembly of an Antarctic sea-ice diatom, *Nitzschia* sp. (most likely *Nitzschia stellata*), and examine its genomic characteristics using comparative analyses. We found that *Nitzschia* sp. and *Fragilariopsis cylindrus*, another Antarctic sea-ice diatom, share numerous orthologous genes that are absent from temperate diatoms. These shared genes are associated with biological processes such as oxidative stress response, DNA repair, protein quality control, osmotic regulation, and nutrient acquisition and transport—functions that may be relevant for coping with the combined stresses of the sea-ice environment. In addition, *Nitzschia* sp. shows contraction in several gene families, including those involved in proprotein convertase activity, clathrin-mediated endocytosis, BTB/POZ domain-containing proteins, solute carrier transporters, and voltage-gated potassium channels. We also identified a high-quality endogenous viral element (EVE) related to the viral family *Metaviridae*, as well as a high proportion of transposable elements (TEs), accounting for 27.25% of the genome assembly. Together, our results characterize the genomic features of *Nitzschia* sp. and highlight potential genomic strategies that may contribute to survival in sea-ice environments.

## Graphical Abstract

**Figure:**
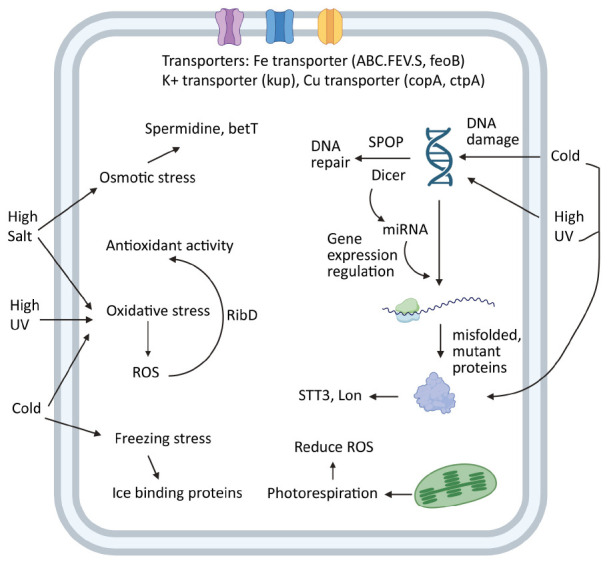
Potential evolutionary adaptative mechanisms in the Antarctic sea-ice diatom *Nitzschia* sp. for coping with extreme environmental stress. Ice-binding proteins mitigate freezing damage, and antioxidant-related proteins (e.g., RibD) protect against oxidative stress. Osmoprotectants such as spermidine and betT help maintain osmotic balance. Specialized transporters facilitate the uptake of essential nutrients, including Fe, K, and Cu. Genomic stability is supported by proteins like SPOP and Dicer, which aid in DNA repair, and STT3 and Lon, which ensure protein quality control. Additionally, photorespiration contributes to ROS scavenging.

## 1 Introduction

Sea ice, covering approximately 7% of the global ocean, represents one of the Earth’s largest biomes (Weeks, 2010). It serves as a critical habitat for many organisms, including primary producers that thrive either attached to the ice or within its brine inclusions. In the Antarctic, primary production within sea ice is estimated to range from 23 to 70 Tg C per year, accounting for about 12% of the total production in the Antarctic sea-ice zone (Legendre et al., 1992; Arrigo et al., 1997; Saenz and Arrigo, 2014). Diatoms dominate these algal communities, forming the foundation of the Antarctic sea-ice ecosystem. These diatoms play an essential role in transferring energy and nutrients through Antarctic marine food webs, supporting diverse organisms, including krill—a keystone species in the Antarctic ecosystem (Schmidt et al., 2014). Moreover, diatoms significantly contribute to biogeochemical cycles via the export of organic carbon to deeper ocean layers during seasonal ice melt (Vancoppenolle et al., 2013; Duncan and Petrou, 2022).

The sea-ice environment is characterized by extreme and highly dynamic conditions, including sub-zero temperatures, elevated salinities, restricted gas exchange, and strongly fluctuating light regimes (Thomas and Dieckmann, 2002). To persist under these pressures, sea-ice algae have evolved diverse genomic and physiological strategies that enhance tolerance to cold, osmotic stress, and oxidative damage (Mock et al., 2017; Kennedy et al., 2019; Zhang et al., 2020; Zhang et al., 2023a). Genomic studies of Antarctic sea-ice green algae, such as *Microglena* sp. YARC and *Chlamydomonas* sp. ICE-L, have provided important insights into potential adaptive mechanisms, including gene family expansion, horizontal gene transfer, and enhanced capacities for photoprotection, lipid metabolism, and DNA repair (Zhang et al., 2020; Zhang et al., 2023a). However, these taxa are relatively minor components of Antarctic sea-ice algal assemblages. In contrast, diatoms dominate Antarctic sea-ice algal communities and represent some of the most ecologically abundant and productive microorganisms in polar oceans (Kang and Fryxell, 1992). Among them, *Fragilariopsis cylindrus* has emerged as a model species, with genomic and transcriptomic analyses revealing extensive allelic divergence and environmentally responsive gene regulation under stressors such as iron limitation, prolonged darkness, freezing temperatures, and elevated CO*2* (Mock et al., 2017; Kennedy et al., 2019; Joli et al., 2024). These findings underscore the central role of diatoms in Antarctic sea ice and highlight the importance of expanding genomic investigations beyond a single model species.

Despite the ecological dominance of diatoms in Antarctic sea ice, our understanding of their genomic adaptation remains limited, with most available studies focused on *F. cylindrus*. Here, we present a high-quality metagenome-assembled genome (MAG) of *Nitzschia* sp. (most likely *Nitzschia stellata*), recovered from Antarctic fast ice in McMurdo Sound. *Nitzschia* species are well-documented as dominant members of bottom sea-ice diatom assemblages in this region, and *N. stellata* in particular has been repeatedly reported as one of the most abundant taxa in McMurdo Sound sea ice (Martin et al., 2012; McMinn et al., 2010; Kennedy et al., 2020). Consistent with these observations, *Nitzschia* sp. emerged as the most complete and abundant diatom MAG recovered from our sea-ice metagenome, motivating its selection for downstream genomic analyses. By integrating comparative genomic analyses of *Nitzschia* sp. and *F. cylindrus*, we aim to broaden the current genomic perspective on Antarctic sea-ice diatoms and to explore shared and lineage-specific genomic features that may underpin their persistence in polar sea-ice environments.

## 2 Materials and methods

### 2.1 Sampling, DNA extraction, sequencing

Sea-ice algae were sampled from the bottom 300 mm of 1.8 m thick land-fast (fast) ice at Cape Evans, McMurdo Sound, Antarctica (77°38’S, 166°24’E) in November 2022. Cape Evans lies on the western coast of Ross Island in southern McMurdo Sound, in a region characterized by seasonal fast ice and high ice-associated primary productivity. Fast ice at this site typically forms during austral autumn and persists through spring, providing a stable substratum for the development of dense under-ice algal communities. *N. stellata*, *Berkeleya adeliensis*, *Fragilariopsis* spp.*, Navicula* spp. have been documented as dominant diatoms in bottom ice around McMurdo Sound (Martin et al., 2012; McMinn et al., 2010; Kennedy et al., 2020). At the time of our sampling, fast ice exhibited visible brownish ice algal biomass at the ice–water interface, indicative of substantial under-ice diatom communities. The site was selected as part of an ongoing McMurdo Sound sampling program and not exclusively based on *Nitzschia* dominance, although *Nitzschia* spp. are known to be common components of Antarctic bottom ice diatom assemblages in this region. Sea ice cores were extracted using a Mark V SIPRE corer (Kovaks, United States) and immediately transferred to a field laboratory, where they were melted naturally at 4°C overnight without the addition of seawater. The melted sea ice was sequentially filtered through a 20 μm mesh followed by 0.2 μm pore-size polycarbonate (PC) membrane filters (Millipore, GTTP14250) using a peristaltic pump. The initial 20 μm pre-filtration step was applied to remove large particles and debris prior to microbial collection. The 0.2 μm filters were flash-frozen in liquid nitrogen and stored at −80°C until DNA extraction. It should be noted that this filtration protocol was designed primarily for the collection of microbial communities and is therefore not optimal for comprehensive algal sampling, as the 20 μm pre-filtration step removes large diatom cells. In contrast, algal-focused sampling typically employs a size-fractionated filtration strategy, such as sequential filtration through a 200 μm mesh to remove zooplankton, followed by 20 μm filters to retain large algae and 3 μm filters to collect smaller algal cells. Total DNA on filters was extracted using the Water DNA Kit (Omega) according to the manufacturer’s instructions. Library construction was performed using a NEBNext Ultra DNA Library Prep Kit (New England Biolabs, Ipswich, MA, United States), and high-throughput sequencing of the total DNA was conducted using the Illumina NovaSeq 6000 (paired-end sequencing, 2 × 150 bp) platform (Novogene Bioinformatics Technology Co., Ltd., Nanjing, China).

### 2.2 Diatom genome assembly and phylogenetics

The raw sequencing data were quality-controlled by the sequencing provider using Trimmomatic v0.36 (Bolger et al., 2014) and subsequently assembled using MEGAHIT v1.2.9 with the default k-mer series (Li et al., 2015). Contigs were classified using Tiara v1.0.3 with default parameters, a deep-learning-based tool designed to identify eukaryotic sequences in metagenomic datasets (Karlicki et al., 2022). Contigs classified as eukaryotic, organellar, or unknown were retained for subsequent binning. Binning was performed by metaBAT2, CONCOCT, and MaxBin2 in metaWRAP v1.3 (–concoct –maxbin2 –metabat2 –universal) (Uritskiy et al., 2018). Genome assembly completeness of bins was assessed using BUSCO v5.8.0 (Benchmarking Universal Single-Copy Orthologs) with parameters “–miniprot” and “–auto-lineage-euk” (Simão et al., 2015), and bins with genome completeness > 90% were initially classified as high quality. Among the three binning approaches, metaBAT2 produced the highest-quality bins for this eukaryotic metagenome, as indicated by higher BUSCO completeness scores. Bin dereplication was performed using dRep v3.4.2 (parameter: dRep compare) (Olm et al., 2017). High-quality diatom bins were subsequently curated and visualized using BlobToolKit on the Galaxy platform (Challis et al., 2020), during which contigs taxonomically inconsistent with the dominant lineage of the bin (i.e., diatoms) were removed to minimize contamination. The BlobToolKit workflow on Galaxy followed the tutorial provided at Galaxy Training Network. The taxonomy of the diatom genome was initially assigned using the Bin Annotation Tool v5.2.3 (BAT) on the Galaxy platform with default settings (Von Meijenfeldt et al., 2019), and a finer taxonomic resolution was subsequently obtained through phylogenetic tree reconstruction. The phylogenetic tree was constructed following a slightly modified version of the Snakemake workflow described by Manni et al. (2021). Briefly, first, reference genome assembly and corresponding GFF files of lineage-related diatoms (representative *Bacillariophyceae* diatoms) were downloaded from NCBI datasets. Then, orthologous genes of these diatoms were identified using BUSCO, and proteins from each orthologous group were aligned with MAFFT (Katoh and Standley, 2013) and trimmed using trimAl (Capella-Gutiérrez et al., 2009). The resulting alignments were concatenated with AMAS (Borowiec, 2016), and a maximum-likelihood phylogeny was inferred from the concatenated superalignment using IQ-TREE 2 (Minh et al., 2020). In our modified workflow, parameters in the *config.yaml* file were adjusted as follows: “path_to_genomes” was set to “genomes,” “keep_duplicates” was set to “Yes,” and “dataset” was specified as “stramenopiles_odb10.” Given the high prevalence of duplicated genes in diatom genomes, duplicated BUSCO genes (“keep_duplicates” = “Yes”) were retained to ensure sufficient phylogenetic signal for tree reconstruction. All tools were run with the authors’ default parameters, and the workflow was executed using the command “snakemake –cores 16 –use-conda.” The visualization of the phylogenetic tree was performed by iTOL (Letunic and Bork, 2024).

### 2.3 Gene annotation and gene family analysis

Protein-coding genes in the *Nitzschia* sp. genome were predicted using MAKER2 v2.31.11 on the Galaxy platform (Holt and Yandell, 2011; The Galaxy Community, 2024), following the protocol described at the Galaxy Training Network. Within the MAKER2 workflow, expressed sequence tags (ESTs) from the lineage-related diatom species (*F. cylindrus*) were downloaded from GenBank, and the protein reference database was obtained from OrthoDB v12 (Tegenfeldt et al., 2025). The MAKER2 workflow produced a final annotation in GFF format, from which exon, CDS, and protein sequences were extracted using GFFread (Pertea and Pertea, 2020). Exon sequences were used as input for BUSCO to assess the quality of the MAKER2 gene annotation, with BUSCO run in *transcriptome* mode using the *stramenopiles_odb10* dataset. Gene annotation quality was considered good when transcriptome BUSCO completeness was comparable to, or only slightly lower than, genome BUSCO completeness, indicating that the predicted gene models adequately captured the conserved gene content of the genome. Comparative genomic analyses were performed using OrthoVenn3, an integrated platform for whole-genome comparison, annotation, and evolutionary analysis (Sun et al., 2023). Eight *Bacillariophyceae* genomes were selected to enable a focused comparison between Antarctic sea-ice–associated diatoms and non–sea-ice diatoms from temperate or tropical environments. The two Antarctic sea-ice diatoms included were *Nitzschia* sp. (this study) and *F. cylindrus*. The six non–sea-ice diatoms (*Seminavis robusta*, *Nitzschia inconspicua*, *Cylindrotheca closterium*, *Fistulifera solaris*, *Phaeodactylum tricornutum*, and *Pseudo-nitzschia multistriata*) represent well-characterized model or reference diatom species isolated from coastal, benthic, or open-ocean environments in temperate or tropical regions. These species were selected based on the availability of high-quality reference proteomes, which are required for reliable orthologous gene clustering in OrthoVenn3. For all comparative species except *Nitzschia* sp., reference proteomes were obtained from UniProt and used as inputs for the OrthoVenn3 workflow. Within OrthoVenn3, orthologous clustering was performed with OrthoMCL with inflation value of 1.5, phylogenetic reconstruction with FastTree2 under the LG + CAT model, and gene family expansion and contraction analyses with CAFE5, all using default parameters. Gene family annotation and functional prediction for shared unique, expanded, and contracted gene sets in *Nitzschia* sp. and *F. cylindrus* were conducted using InterPro and GhostKOALA (Blum et al., 2024; Kanehisa et al., 2016).

### 2.4 Endogenous viral elements and transposable elements

Endogenous viral elements (EVEs) in the *Nitzschia* sp. genome were analyzed using the “Viruses and Plasmids” workflow on the NMDC platform. This workflow detects viral and plasmid sequences in assembled scaffolds using geNomad (Camargo et al., 2024) and evaluates viral genome quality with CheckV (Nayfach et al., 2021). The workflow was executed using *default* parameters, and high-quality EVEs were defined as those with CheckV-estimated completeness > 90%. Predicted protein-coding genes within the high-quality EVE were initially generated by the NMDC workflow. To further ensure comprehensive gene prediction given the structural complexity and evolutionary divergence of EVEs, additional gene prediction was performed using Prodigal v2.6.3 (Hyatt et al., 2010) and NCBI ORF Finder. Both tools were run using genetic codes 1 (standard) and 11 (bacterial), allowing alternative start codons, with a minimum ORF length of 150 nt. This dual-genetic code approach was applied to account for potential heterogeneity in coding signals arising from viral origins and host integration. All predicted proteins were functionally annotated using InterPro (Blum et al., 2024), with all available member databases and sequence feature options enabled. The phylogenetic tree of the high-quality EVE was constructed using ViPTree, which infers proteomic trees based on normalized tBLASTx similarity scores (SG) and genomic distances (1-SG) using the BIONJ algorithm (Nishimura et al., 2017). For tree construction, ssRNA-RT reference viruses from the built-in ViPTree database, together with representative *Ortervirales* viruses downloaded from GenBank, were included to infer the phylogenetic placement of the high-quality EVE. The existence of long terminal repeats (LTR) in the high-quality EVE was checked by LTR_retriever v3.0.4 (Ou and Jiang, 2018). Transposable elements (TEs) within the eight diatom genomes were predicted by RepeatModeler v2.0.5 (Flynn et al., 2020). The classification and proportion of TEs in each diatom were identified and calculated using RepeatMasker v4.1.5. These tools were all run with default parameters.

## 3 Results

### 3.1 Diatom genome, phylogenetics, and genes

**FIGURE 1:**
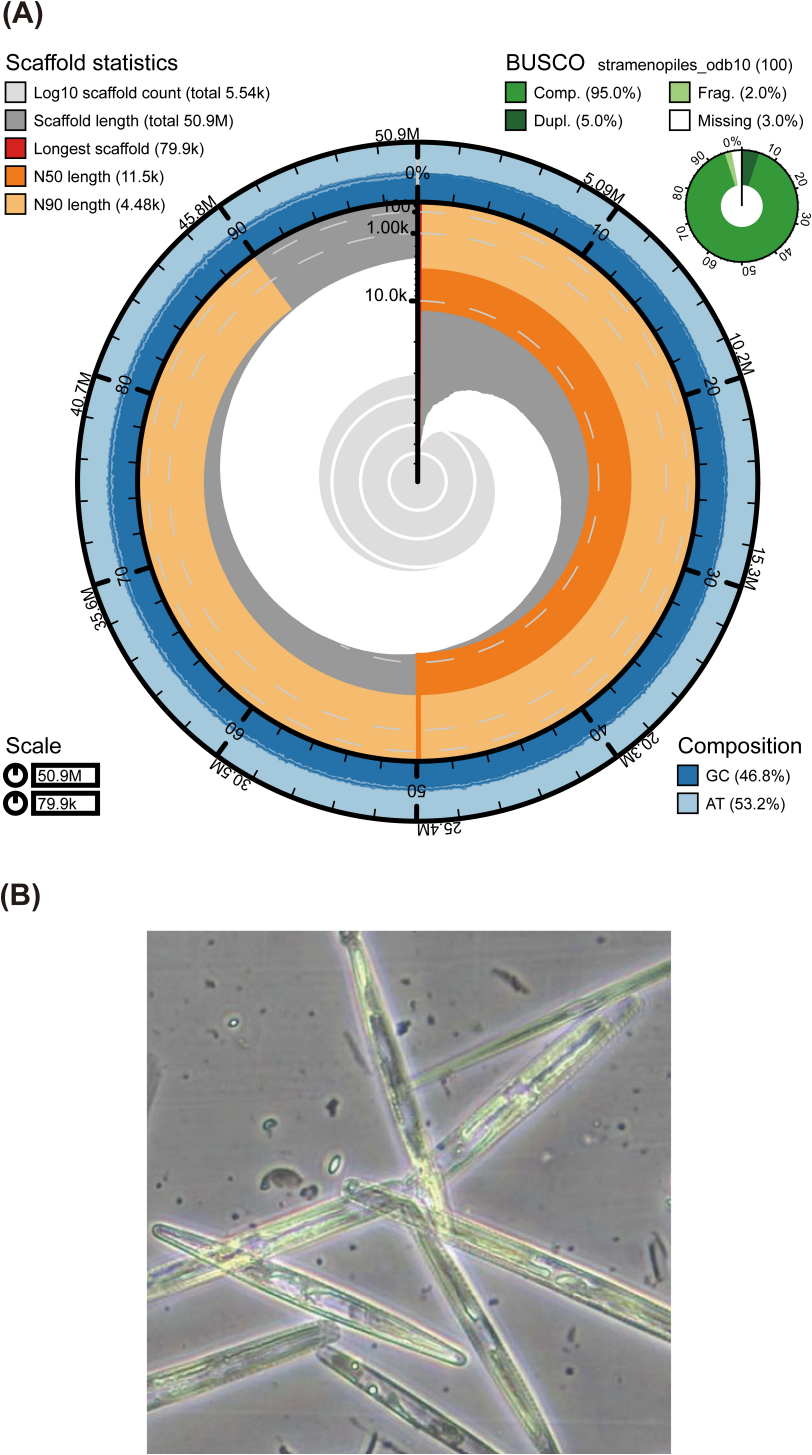
Genome assembly and morphology of *Nitzschia* sp. (most likely *Nitzschia stellata*). **(A)** Snail plot of the *Nitzschia* sp. genome assembly (50.9 Mb). The longest scaffold is shown in red, while orange and light-orange arcs represent the N50 and N90 scaffold lengths, respectively. GC and AT contents distributions are depicted in dark blue and blue. BUSCO results in the upper right indicate genome completeness, including the proportions complete (Comp.), duplicated (Dupl.), fragmented (Frag.), and missing orthologs. **(B)** Light microscopy image of *N. stellata* cells. Cells occur either singly or attached end-to-end, forming straight, zig-zag, or stellate colonies of 3–12 cells. Valves are linear to lanceolate in valve view, tapering toward the poles, with an apical axis of 60–134 μm and a transapical axis of 6.5–10.5 μm.

**FIGURE 2:**
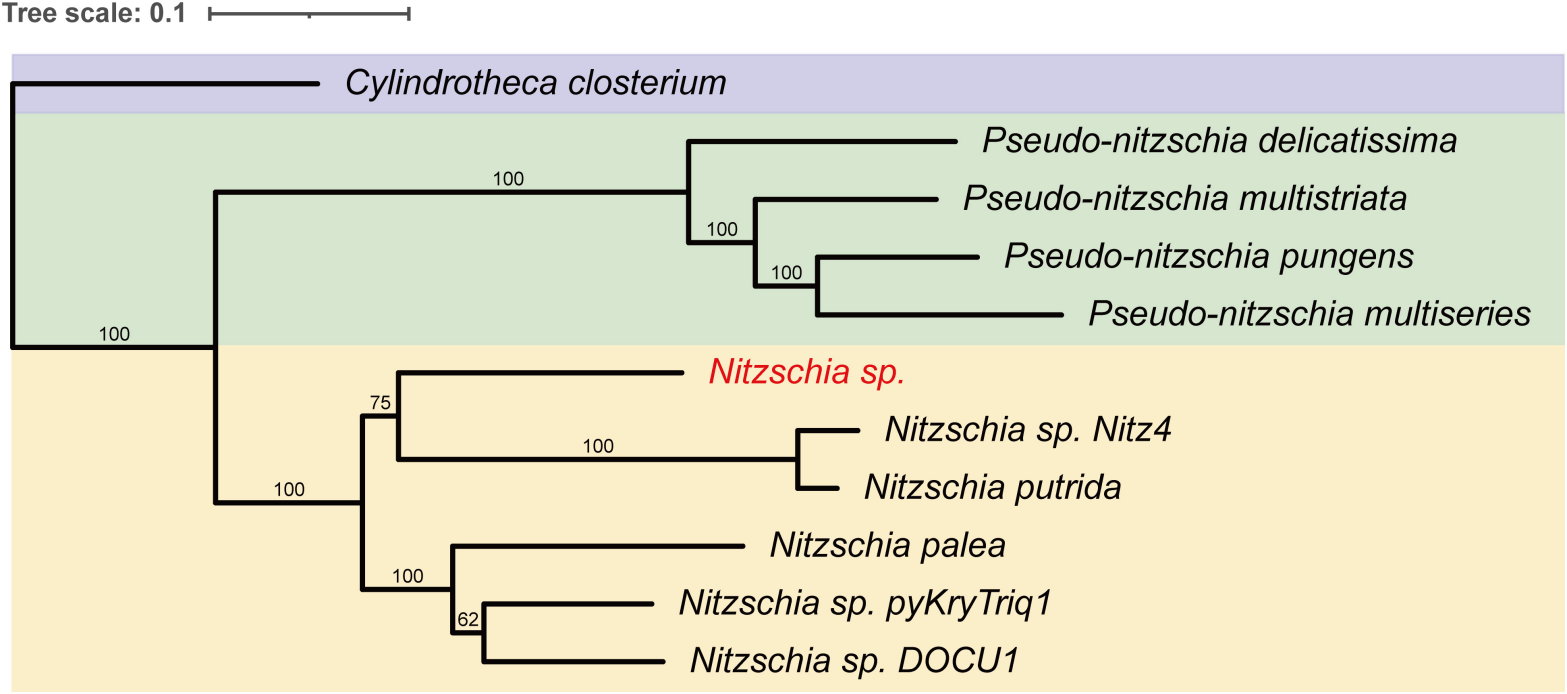
Maximum likelihood phylogenetic tree of the *Nitzschia* sp. metagenomic assembled genome (MAG) and ten additional *Bacillariaceae* diatom genomes, inferred from a concatenated alignment of the 24 orthologous genes. *Cylindrotheca closterium* was used as the outgroup species. Bootstrap support values are shown at each node. Clades are color-coded: purple for *Cylindrotheca*, green for *Pseudo-nitzschia*, and yellow for *Nitzschia*.

A draft MAG of a diatom was recovered from Antarctic sea-ice metagenomic data, yielding a total genome size of 50.9 Mb with an N50 of 11.5 kb and a GC content of 46.8% (Figure 1A). BUSCO analysis indicated a 95% genome completeness, denoting a high-quality MAG assembly (Figure 1A). Taxonomic annotation using BAT classified this MAG within the family *Bacillariaceae*, and phylogenetic reconstruction placed it within the genus *Nitzschia* (Figure 2). Microscopic identification suggested the species was *N. stellata*, a dominant diatom found in our sea ice samples and in previous sea ice collections (Martin et al., 2012; Ryan et al., 2009; McMinn et al., 2010; Kennedy et al., 2020; Supplementary Table 1). *N. stellata* cells were observed as solitary or attached end-to-end, forming straight, zig-zag, or stellate colonies comprising 3–12 cells; valves were linear to lanceolate in valve view, tapering toward the poles, with an apical axis of 60–134 μm and a transapical axis of 6.5–10.5 μm (Figure 1B; Scott and Marchant, 2005). For clarity, this diatom MAG was hereafter referred to as *Nitzschia* sp. Gene prediction using MAKER2 identified 22,781 protein-coding genes in *Nitzschia* sp. A subsequent BUSCO assessment of the predicted transcripts confirmed a 95% completeness, supporting the accuracy of the gene annotation.

### 3.2 Comparative genomic analysis

**FIGURE 3:**
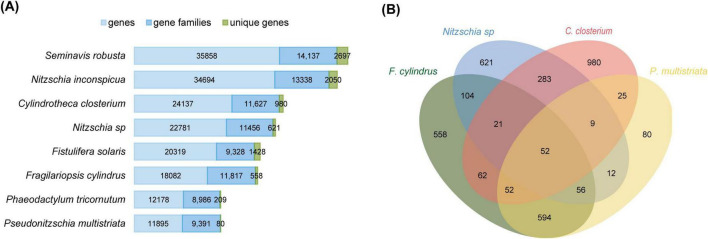
Comparative gene family analysis of *Nitzschia* sp. **(A)** Predicted gene counts, gene family numbers, and unique gene families for eight *Bacillariophyceae* diatom genomes. Sky blue bars represent the total number of predicted genes (annotated using MAKER2), blue bars denote the number of gene families, and green bars indicate unique gene families. Gene family inference was conducted using OrthoVenn3. **(B)** Numbers of shared and lineage specific gene families identified in four *Bacillariophyceae* diatoms. Gene family counts were inferred with OrthoVenn3. The Antarctic sea-ice isolates *Nitzschia* sp. and *F. cylindrus* are phylogenetically most closely related to *C. closterium* and *P. multistriata*, respectively.

Orthologous clustering identified 11,456 gene families in *Nitzschia* sp., including 621 gene families unique to this species when compared to seven other diatoms analyzed (Figure 3A). In comparison, *F. cylindrus* possesses 11,817 gene families, with 558 being species-specific (Figure 3A). Notably, *Nitzschia* sp. and *F. cylindrus* share 104 gene families absent in the other six non-polar species analyzed (Figure 3B). Based on KEGG annotations and manual literature searches, these unique genes are associated with diverse biological functions, including oxidative stress response, DNA repair, protein quality control, osmotic homeostasis, and nutrient uptake and transport (Table 1 and Supplementary Tables 2, 3).

**TABLE 1:** Functional annotation of selected unique proteins shared between *Nitzschia* sp. and *F. cylindrus* (column 2), and selected expanded proteins in *Nitzschia* sp. (column 3).

Function	Unique proteins	Expanded proteins
Oxidative stress response	Spermidine synthase (K00797)	RibD* (K11752)
		SHMT* (K00600)
DNA repair, protein quality control	Chaperonin GroEL (K04077)	SMARCA3* (K15711)
	STT3 (K07151)	HMGB (K10802, K11295, K11296)
	SPOP (K10523)	IARS (K01870)
	Endoribonuclease Dicer (K11592)	ATP-dependent Lon protease* (K01338)
	SUPT16H (K25639)	ClpS (K06891)
Osmotic homeostasis	Spermidine synthase (K00797)	STP (K24193)
	Kup (K03549)	BetT (K02168)
Nutrient acquisition and transport	ABC.FEV.S (K02016)	STP (K24193)
	Kup (K03549) P-type Cu + transporter (K17686)	Ferrous iron transport protein B (K04759)

Transcriptional and translational regulation	ABC-F (K06158)	HMGB (K10802, K11295, K11296)
	Endoribonuclease Dicer (K11592)	TUT (K13291)
	SUPT16H (K25639)	BED zinc finger (K24637)
		SSRP1 (K09272)
Others	Ice-binding proteins (pfam: PF11999, PF20597)	

Proteins marked with an asterisk (*) are also expanded in *F. cylindrus*. The corresponding KEGG or Pfam identifiers are provided for each protein.

**FIGURE 4:**
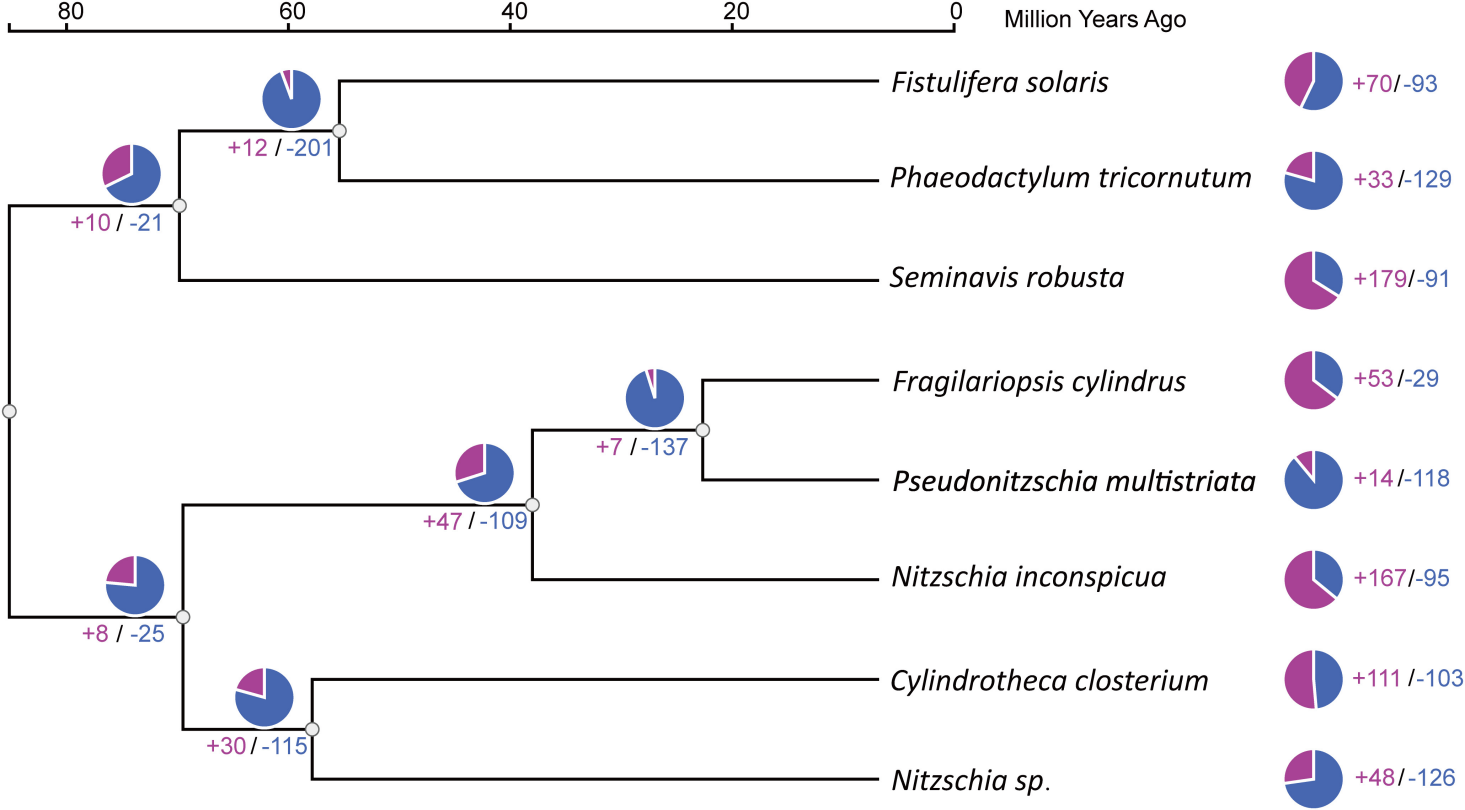
Gene family expansions and contractions across eight *Bacillariophyceae* diatom genomes inferred using CAFE5. Values along branches or beside terminal taxa indicate the number of gene families gained (+) or lost (-) relative to the ancestral node. Gene family expansions are shown in red and contractions in blue.

The analysis of gene family expansions and contractions showed that *Nitzschia* sp. has 48 expanded and 126 contracted gene families (Figure 4). In contrast, *F. cylindrus* shows 53 expanded and 29 contracted gene families (Figure 4). The gene families expanded in both *Nitzschia* sp. and *F. cylindrus* are primarily associated with oxidative stress response, DNA repair, and protein quality control (Table 1). In *Nitzschia* sp., the expanded gene families additionally include those related to transcriptional and translational regulation, DNA repair, protein quality control, and osmotic homeostasis (Table 1 and Supplementary Tables 4, 5). Conversely, the contracted gene families in *Nitzschia* sp. encode proteins such as proprotein convertase subtilisin/kexin proteases, components of clathrin-mediated endocytosis, BTB/POZ domain-containing proteins, members of the solute carrier (SLC) family, and voltage-gated potassium channels (Table 2 and Supplementary Tables 6, 7).

**TABLE 2:** Functional annotation of selected contracted proteins in *Nitzschia* sp.

Function	Proteins
Proprotein convertase subtilisin/kexin proteases (PCSK)	PCSK1 (K01359), PCSK5 (K08654), PCSK6 (K08672), PCSK7 (K08673)
Clathrin-mediated endocytosis	AP2S1 (K11827), LRP1 (K04550), LRP1B (K20049), PIP5K (K00889), SHKBP1 (K21953)
BTB/POZ domain-containing proteins	KCTD1_15 (K21754), KCTD3 (K21915), KCTD7_14 (K21917), KCTD8_12_16 (K21918), KCTD18 (K21921), KCTD21 (K21922)
Solute carrier family (SLC)	SLC4A1 (K06573), SLC4A2 (K13855), SLC4A3 (K13856), SLC4A10 (K13861), SLC34A (K14683), SLC47A (K03327)
Voltage-gated potassium channels	KCNC3 (K04889), KCNK16 (K04924), KCNKF (K05389), KCNJ2 (K04996), KCNJ4 (K04998), KCNJ6 (K05000), KCNJ9 (K05002)

The corresponding KEGG identifiers are provided for each protein.

### 3.3 Endogenous viral elements and transposable elements

**FIGURE 5:**
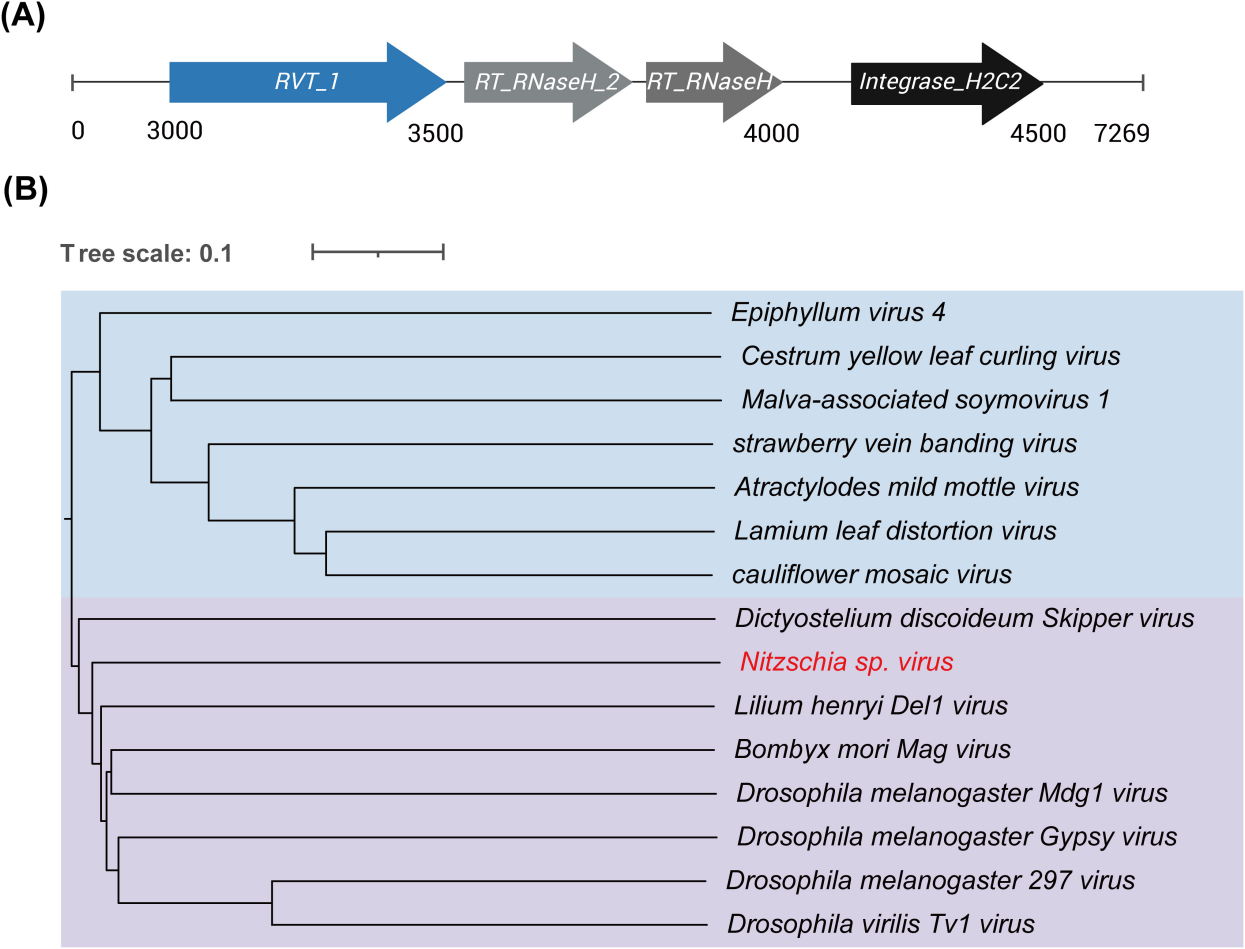
**(A)** Genome map of the high-quality EVE in *Nitzschia* sp. The putative genes were predicted by genomad and annotated by InterPro. **(B)** Proteomic phylogenetic tree of the EVE identified in *Nitzschia* sp., inferred together with representative viruses from the order *Ortervirales* using ViPTree. The tree was constructed based on genome-wide proteomic similarity, with normalized tBLASTx scores used to calculate genomic distances (1-SG) and tree inference performed using the BIONJ algorithm. The placement of the EVE is supported by its consistent clustering with reference viral families. Color-coded clades denote viral families: blue for *Caulimoviridae* and purple for *Metaviridae*.

Forty-six putative EVEs were identified within the *Nitzschia* sp. genome using geNomad, the majority of which were incomplete retroviral-like fragments. One high-quality EVE was identified within a 7,269 bp contig and was classified by CheckV as high-quality with an estimated completeness of 100% (AAI-based, medium confidence). Gene predictions revealed that this element encodes a retrovirus-related Pol polyprotein containing conserved domains, including RVT_1 (reverse transcriptase), RT_RNaseH, RT_RNaseH_2, and Integrase_H2C2 (Figure 5A), which are essential and diagnostic components of reverse-transcribing viruses and retroelements. Phylogenetic analysis based on proteomic similarity placed this element within the *Metaviridae* family (i.e., Ty3/Gypsy-like LTR retroelements) (Figure 5B). Notably, although canonical *Metaviridae* elements typically encode gag/capsid proteins and long terminal repeats (LTRs), these features were not annotated in this EVE. Importantly, the CheckV completeness estimate here reflects the recovery of a near-complete Pol region, rather than the presence of all structural modules, which may be absent, highly diverged, or unrecognizable in endogenous viral elements. Furthermore, a high proportion of TEs (27.25% of the total genome size) was detected in *Nitzschia* sp., with LTR elements, particularly Ty1/Copia, being the most abundant (Supplementary Table 8). Among the eight diatom genomes analyzed, the proportion of TEs varied considerably, with no clear correlation to geographical distribution (i.e., polar vs. non-polar) (Supplementary Table 8).

## 4 Discussion

### 4.1 Genomic adaptations of two Antarctic sea-ice diatoms

The Antarctic sea ice environment is characterized by freezing temperatures, high salinity, and extreme light conditions, all of which impose oxidative, osmotic, and freezing stresses on resident microbial communities. Comparative genomic analyses here show that Antarctic sea-ice diatoms, including *Nitzschia* sp. and *F. cylindrus*, share a subset of expanded or unique gene families relative to six non-polar diatom species (Tables 1, 2). These genes suggest several candidate functional pathways that may be relevant to survival and thrive in sea-ice environments. Oxidative stress, caused by excessive reactive oxygen species (ROS) production, can damage lipids, proteins, and nucleic acids, ultimately impairing cellular function and viability (Kennedy et al., 2020). Results presented here show that both two sea-ice diatoms have expanded gene families encoding RibD (KEGG: K11752) and SHMT (KEGG: K00600), which may be help mitigate the detrimental effects of oxidative stress. RibD participates in riboflavin biosynthesis, and riboflavin has been shown to contribute to cold tolerance in plants (Chen et al., 2006). For instance, riboflavin enhances non-enzymatic antioxidant activity, thereby reducing chilling injury and improving cold tolerance in zucchini fruit (Castro-Cegrí et al., 2024). It also helps regulate energy metabolism in strawberries during cold storage by suppressing respiration rate increases, limiting ROS accumulation, and maintaining soluble sugar content (Zhang et al., 2023b). SHMT (serine hydroxymethyltransferase) is a key enzyme in the photorespiratory pathway, a crucial mechanism for dissipating excess light energy and protecting cells from oxidative damage (Moreno et al., 2005; Yan et al., 2024). Expansion of photorespiration-related genes has also been observed in Antarctic green algae, hypothesizing that sea-ice algae may utilize photorespiration to reduce ROS production and mitigate oxidative damage (Zhang et al., 2019). In addition, both sea-ice diatoms possess a spermidine synthase (KEGG: K00797) and ice-binding protein genes (pfam: PF11999, PF20597), which are absent from six non-polar diatom species. Spermidine synthase produces spermidine, a polyamine that plays an essential role in plant cold acclimation (Alcázar et al., 2011). Spermidine functions as both an osmoprotectant, stabilizing cellular structures under cold stress, and a ROS scavenger that mitigates oxidative damage at low temperatures (Alcázar et al., 2011). It also enhances gene expression and promotes protein repair in cyanobacteria exposed to winter chill-light stress (Zhu et al., 2015). Ice-binding proteins, commonly found in cold-adapted organisms, are thought to have been acquired in Antarctic algae through horizontal gene transfer from bacteria (Zhang et al., 2020). These proteins inhibit ice nucleation and limit crystal growth, thereby reducing freeze-induced damage and osmotic stress (Zhang et al., 2020). Importantly, it should be noted that, the functional interpretations presented in this section are based on comparative genomic patterns rather than direct functional or evolutionary evidence. Owing to the limited availability of functional studies in diatoms and algae, several interpretations are necessarily inferred from higher-plant literature. Moreover, many of the genes discussed here are broadly conserved across eukaryotes and may represent general stress-response capacities rather than polar-specific adaptations. Accordingly, these interpretations are framed as putative hypotheses that require further validation through approaches such as selection analyses, transcriptomic profiling under sea-ice conditions, and experimental functional studies.

The extreme polar environment (e.g., freezing cold, UV exposure) can compromise microbial DNA integrity, disrupt gene expression, and impair protein synthesis. Our results show that *Nitzschia* sp. and *F. cylindrus* share several unique genes (e.g., SPOP, Dicer, STT3) not found in other six non-polar diatoms, which may be contribute to enhanced DNA repair and protein folding capacities (Table 1). SPOP (Speckle-type POZ protein; KEGG: K10523) functions in the DNA damage response (DDR), a coordinated cellular system that detects and repairs genomic lesions (Zhang et al., 2014; Boysen et al., 2015). Dicer (KEGG: K11592), an endoribonuclease, also participates in repairing double-strand breaks (DSBs) (Chen et al., 2017; Yao et al., 2016) and generates small regulatory RNAs (miRNAs) that modulate gene expression and contribute to stress responses (Sunkar et al., 2007; Liu et al., 2008; Lv et al., 2010). STT3 (KEGG: K07151) plays a crucial role in N-glycosylation, a post-translational modification essential for proper protein folding, stability, and function (Koiwa et al., 2003). In *Arabidopsis*, STT3a is a key component of adaptive responses to salt and osmotic stress by promoting the refolding of unfolded proteins (Koiwa et al., 2003), and in jojoba, it may facilitate cold acclimation by enhancing protein folding and quality control in the endoplasmic reticulum (Gao et al., 2019). Additionally, the ATP-dependent Lon protease (KEGG: K01338), which mediates the selective degradation of misfolded or damaged proteins, is expanded in *Nitzschia* sp. and *F. cylindrus* (Table 1). This protease plays vital roles in protein quality control, DNA replication and repair, and diverse stress responses (Lee and Suzuki, 2008; He et al., 2016; Figaj et al., 2020; Kirthika et al., 2023).

*Nitzschia* sp. and *F. cylindrus* also share several unique genes related to nutrient transport and acquisition, including those encoding a P-type Cu^+^ transporter, KUP, and ABC.FEV.S (Table 1). The P-type Cu^+^ transporter (KEGG: K17686) is a membrane-bound ATPase that mediates the translocation of copper ions across cellular membranes. Copper is essential for a range of physiological processes, including photosynthetic and mitochondrial electron transport, oxidative stress management, cell wall metabolism, and hormone signaling (Yruela, 2009). KUP (KEGG: K03549) is a potassium uptake protein that enables efficient K^+^ acquisition, particularly under hyperosmotic stress (Trchounian and Kobayashi, 1999). ABC.FEV.S (KEGG: K02016) is an iron complex transport system substrate-binding protein, which is a key component of the siderophore-dependent iron uptake system, responsible for recognizing and binding iron–ligand complexes (Delepelaire, 2019). Previous studies have also reported an expansion of iron transporters or transferrin genes in Antarctic sea-ice diatoms and green algae (Mock et al., 2017; Zhang et al., 2023a). Furthermore, increased iron availability has been shown to enhance the photosynthetic parameters (Fv/Fm, rETRmax, α) of the sea-ice diatoms *F. cylindrus* and *F. curta* (Pankowski and McMinn, 2009). Thus, it is hypothesized that the expansion of iron transporter genes can enhance iron uptake efficiency, thereby improving photosynthetic performance and supporting the survival of sea-ice diatoms during the polar winter (Pankowski and McMinn, 2009; Zhang et al., 2023a).

### 4.2 Species-specific genomic innovations of *Nitzschia* sp.

In addition to the expanded gene families shared with *F. cylindrus*, *Nitzschia* sp. possesses several unique expansions. These expanded genes, including those encoding a BED zinc finger protein, HMGB proteins, ClpS, IARS, and BetT, are associated with transcriptional regulation, DNA repair, protein quality control, and osmotic homeostasis (Table 1). Collectively, these genes and their associated functional pathways are hypothesized to contribute to the adaptability of *Nitzschia* sp. to sea-ice environments; however, these putative roles require further validation using approaches such as selection analyses, transcriptomic profiling, and targeted functional studies. The BED zinc finger protein (KEGG: K24637) belongs to a novel superfamily of transcription factors that regulate a wide range of processes in plants, including growth, development, and cellular responses to abiotic stresses (Hussain et al., 2022). A related class of zinc finger proteins (C3HC4-type) has also been reported as expanded in Antarctic green algae (Zhang et al., 2023a). This expansion of zinc finger proteins may be attributed to the critical role of zinc in supporting the photosynthetic growth of phytoplankton in low-temperature polar oceans (Ye et al., 2022). HMGB proteins 1, 2, and 3 (KEGG: K10802, K11295, K11296) belong to the High Mobility Group superfamily and function as DNA chaperones, playing a role in various chromatin-associated processes, including transcription, replication, recombination, DNA repair, and stress response (Štros, 2010; Wang and Zhang, 2020). For instance, *HMGB2* and *HMGB3* expression in *A. thaliana* was significantly up-regulated in response to cold stress (Kwak et al., 2007). Isoleucyl-tRNA synthetase (IARS, KEGG: K01870) is an essential enzyme that possesses proofreading mechanisms to remove incorrectly activated amino acids, maintaining the high fidelity of the translation process (Yadavalli and Ibba, 2012). ClpS (KEGG: K06891) is an adaptor protein for the Clp protease (ClpP) that is involved in the degradation of misfolded and dysfunctional proteins, the regulation of short-lived proteins, and stress responses (Guo et al., 2002; Aljghami et al., 2022). The choline/glycine/proline betaine transport protein (betT, KEGG: K02168) functions in the uptake of compatible solutes such as choline, glycine betaine, and proline betaine (Chen and Beattie, 2008). The expansion of this gene is hypothesized to help *Nitzschia* sp. accumulate compatible solutes, thereby maintaining osmotic balance and protecting cellular structures in high-salinity and freezing environments.

*Nitzschia* sp. shows a larger number of inferred gene family contractions (126 vs. 29) than *F. cylindrus*. While this difference may partly reflect lineage-specific evolutionary history or adaptation to sea-ice environments, it is important to emphasize that the *Nitzschia* sp. genome analyzed here represents a draft metagenome-assembled genome, rather than a genome obtained from a clonal axenic culture. Consequently, some inferred gene family contractions may result from assembly fragmentation, uneven sequencing coverage, or binning artifacts, and the apparent absence of genes cannot be unequivocally interpreted as true biological gene loss. To mitigate this limitation, our discussion focuses exclusively on contracted gene families that exhibit functional coherence, in which multiple genes associated with the same biological pathway or cellular process are inferred to be reduced (Figure 4 and Table 2). The likelihood that several independent genes from a single pathway are simultaneously missing due solely to random annotation incompleteness is relatively low, lending increased confidence to these contraction patterns as potential genomic trends rather than isolated artifacts. Nevertheless, these interpretations should be viewed as hypotheses that require validation using more complete genome assemblies derived from clonal cultures and/or functional evidence. Within this framework, several biologically coherent contraction patterns were observed in *Nitzschia* sp. (Table 2). Proprotein convertase subtilisin/kexin-like proteases (PCSKs), including PCSK1 (KEGG: K01359), PCSK5 (KEGG: K08654), PCSK6 (KEGG: K08672) and PCSK7 (KEGG: K08673), which are involved in the processing of various protein precursors (Bae et al., 2008), were inferred to be reduced in *Nitzschia* sp. This reduction may indicate a decreased requirement for processing specific protein precursors or a loss of redundant PCSK functions in *Nitzschia* sp. Additionally, genes encoding proteins involved in clathrin-mediated endocytosis, such as low-density lipoprotein receptor family members LRP1 (KEGG: K04550) and LRP1B (KEGG: K20049), were inferred to be contracted. Clathrin-mediated endocytosis is an essential process for internalizing extracellular molecules, facilitating functions such as neurotransmission, signal transduction, and plasma membrane regulation (McMahon and Boucrot, 2011). Interestingly, *Nitzschia* sp. possesses a distinct LDLR homologue, LRP5_6, which was not identified in the other seven analyzed diatoms. This suggests that *Nitzschia* sp. may employ specialized lineage-specific proteins to fulfill similar cellular functions. Voltage-gated potassium channels, such as KCNC3 (KEGG: K04889), KCNK16 (KEGG: K04924), KCNJ2 (KEGG: K04996), and KCNJ9 (KEGG: K05002), were also inferred to be contracted in *Nitzschia* sp. These channels are crucial for maintaining cell excitability and environmental sensing (Kim and Nimigean, 2016; Hedrich, 2019). The reduced repertoire of voltage-gated K^+^ channels suggest *Nitzschia* sp. may have altered ion transport or environmental signaling requirements.

### 4.3 Endogenous viral elements and transposable elements

Several EVEs and a large number of TEs were identified within the *Nitzschia* sp. genome. EVEs are remnants of viral DNA that have become integrated into the host genome over evolutionary time (Frank and Feschotte, 2017). EVEs, which are widespread in eukaryotes, can drive genomic innovation by donating novel genes and can sometimes act as defensive factors against related viruses (Feschotte and Gilbert, 2012; Veglia et al., 2023). In this study, a high-quality EVE, related to the *Metaviridae* viral family (i.e., Ty3/Gypsy LTR retroelements), was detected in the *Nitzschia* sp. genome. Its relatively high sequence completeness suggests a relatively recent integration or a well-preserved viral relic. At present, however, there is no direct evidence that this EVE is transcriptionally active or functionally relevant, and its presence should therefore be interpreted as a descriptive genomic feature rather than an adaptive trait. Determining whether this EVE has any biological significance will require future transcriptomic and functional analyses. In addition, although gag/capsid proteins and LTRs are typically present in *Metaviridae* viruses, their absence in this EVE is not unexpected. Endogenous viral elements often undergo degeneration, recombination, or structural rearrangement following genomic integration, leading to the loss or divergence of certain viral modules over evolutionary time (Katzourakis and Gifford, 2010; Johnson, 2019). Also, limitations in current gene prediction tools may further hinder the detection of highly diverged gag or LTR sequences in EVEs.

Transposable elements (TEs) make a substantial contribution (27.25%) to the genome assembly of *Nitzschia* sp., which is within the upper range reported for diatom genomes (Zheng et al., 2025). TEs, or “jumping genes,” are mobile DNA segments capable of changing position within a genome, thereby influencing genome rearrangement and expansion (Hermann et al., 2014). In diatoms, LTR retrotransposons are the most abundant class of TEs (Hermann et al., 2014) and the above newly identified high-quality EVE also belongs to this clade. LTR retrotransposons have been implicated in stress responses in both plants and diatoms (Hermann et al., 2014; Pargana et al., 2019; Egue et al., 2015; Maumus et al., 2009). For instance, in the diatom *Leptocylindrus aporus*, LTR retrotransposon transcripts are significantly upregulated under cold stress, with TE-related transcripts linked to antioxidant activity (Pargana et al., 2019). In *P. tricornutum*, the LTR retrotransposon *Surcouf*, which contains regulatory motifs such as heat-shock elements, stress-response elements and CCAAT boxes, is strongly overexpressed under heat stress in parallel with small heat-shock proteins (Egue et al., 2015). Moreover, in *P. tricornutum*, LTR retrotransposons display increased transcriptional activity under nitrate limitation and in response to diatom-derived reactive aldehydes, which can trigger stress responses and cell death (Maumus et al., 2009). However, our study did not find a clear relationship between overall TE abundance and geographical distribution (i.e., polar versus non-polar diatoms). This lack of correlation suggests that TE accumulation alone is unlikely to be a driver of adaptation to polar or sea-ice environments. Instead, previous studies indicate that TE regulation, transcriptional activation, and specific TE lineages may be more relevant for stress responses (Maumus et al., 2009; Egue et al., 2015; Pargana et al., 2019). As transcriptional activity and epigenetic regulation of TEs were not assessed in the present study, no direct functional or adaptive role can be inferred here. Future transcriptomic and experimental studies will be necessary to determine whether TEs in *Nitzschia* sp. and other sea ice diatoms are stress-responsive or contribute to genome plasticity under sea-ice conditions.

In conclusion, this study presents a draft MAG of an Antarctic sea-ice diatom, *Nitzschia* sp., providing a genomic resource for exploring potential mechanisms underlying life in extreme polar environments. Comparative analyses reveal that *Nitzschia* sp. shares a number of unique or expanded genes with *F. cylindrus*, including genes associated with oxidative stress responses, DNA repair, protein quality control, osmotic regulation, and nutrient acquisition and transport. While these genes are broadly conserved across eukaryotes, their shared presence or expansions in sea-ice diatoms highlights functional capacities that may be relevant for coping with the combined stresses of the sea-ice habitat. In addition, *Nitzschia* sp. exhibits pronounced contraction of several gene families, including those related to proprotein convertase subtilisin/kexin proteases, clathrin-mediated endocytosis, BTB/POZ domain-containing proteins, solute carrier transporters, and voltage-gated potassium channels. We also identified a high-quality EVE related to the viral family *Metaviridae*, as well as a substantial proportion of transposable elements (27.25% of the genome assembly). While no direct adaptive role can be inferred from these observations, they document notable features of genome composition and potential genome plasticity in this Antarctic diatom. Overall, this study characterizes the genomic features of *Nitzschia* sp. and provides a comparative framework for future studies. Further work integrating population genomics, transcriptomics, and experimental approaches will be necessary to determine how these genomic patterns contribute to ecological success and persistence of diatoms in Antarctic sea-ice ecosystems.

## Data Availability

The data presented in this study are publicly available in the NCBI (https://www.ncbi.nlm.nih.gov/), accession PRJNA1454863.
