# Supplementary material for: Comparative genomics of an Antarctic sea-ice diatom (Nitzschia sp.) provides insights into potential polar adaptation
Source: Front Microbiol. 2026 Apr 23;17:1755917. doi: 10.3389/fmicb.2026.1755917 (PMC13149256; doi:10.3389/fmicb.2026.1755917)
Supplement: Supplementary file 1 [file Table_1.docx]

Supplementary Material

# Supplementary Tables

Table 1. Relative abundance of diatom species identified in sea ice samples.

| Diatom species | Replicate 1 (%) | Replicate 2 (%) |
| --- | --- | --- |
| *Nitzschia stellata* | 72.20216606 | 71.68458781 |
| *Bekeleya adaliense* | 20.57761733 | 16.84587814 |
| *Nvaicula glaciei* | 2.527075812 | 7.168458781 |
| *Entomeneis Kjellmanni* | 2.527075812 | 1.792114695 |
| *Nitschia lecointei* | 1.444043321 | 1.792114695 |
| *Fragilariaopsis curta* | 0.722021661 | 0.716845878 |

Table 2. Unique protein/domain families shared between *Nitzschia* sp. and *F. cylindrus*, annotated using Pfam.

| Pfam no. | Pfam annotation |
| --- | --- |
| PF10354 | rRNA (uridine-N3-)-methyltransferase BTM5-like |
| PF01494 | FAD binding domain |
| PF00917 | MATH domain |
| PF13561 | Enoyl-(Acyl carrier protein) reductase |
| PF13407 | Periplasmic binding protein domain |
| PF03372 | Endonuclease/Exonuclease/phosphatase family |
| PF00005 | ABC transporter |
| PF11999 | Ice-binding-like |
| PF13426 | PAS domain |
| PF00581 | Rhodanese-like domain |
| PF00595 | PDZ domain |
| PF00254 | FKBP-type peptidyl-prolyl cis-trans isomerase |
| PF00571 | CBS domain |
| PF00564 | PB1 domain |
| PF12848 | ABC transporter |
| PF10152 | Subunit CCDC53 of WASH complex |
| PF00884 | Sulfatase |
| PF13966 | zinc-binding in reverse transcriptase |
| PF00300 | Histidine phosphatase superfamily (branch 1) |
| PF12680 | SnoaL-like domain |
| PF04982 | HPP family |
| PF00403 | Heavy-metal-associated domain |
| PF02675 | S-adenosylmethionine decarboxylase |
| PF01564 | Spermine/spermidine synthase domain |
| PF08238 | Sel1 repeat |
| PF05721 | Phytanoyl-CoA dioxygenase (PhyH) |
| PF01497 | Periplasmic binding protein |
| PF04755 | PAP_fibrillin |
| PF00646 | F-box domain |
| PF13462 | Thioredoxin |
| PF20597 | Putative Ice-binding-like adhesive domain |
| PF13616 | PPIC-type PPIASE domain |
| PF13275 | S4 domain |
| PF00639 | PPIC-type PPIASE domain |

Table 3. Unique protein families shared between *Nitzschia* sp. and *F. cylindrus*, annotated using KEGG.

| KEGG no. | KEGG annotation |
| --- | --- |
| K17686 | copA, ctpA, ATP7; P-type Cu+ transporter |
| K10523 | SPOP; speckle-type POZ protein |
| K11481 | AURKA; aurora kinase A |
| K00797 | speE, SRM, SPE3; spermidine synthase |
| K21806 | VCPKMT, METTL21D; protein N-lysine methyltransferase METTL21D |
| K15738 | uup; ABC transport system ATP-binding/permease protein |
| K02016 | ABC.FEV.S; iron complex transport system substrate-binding protein |
| K06917 | selU, mnmH; tRNA 2-selenouridine synthase |
| K10457 | KLHL20, KLEIP; kelch-like protein 20 |
| K06968 | rlmM; 23S rRNA (cytidine2498-2'-O)-methyltransferase |
| K01902 | sucD; succinyl-CoA synthetase alpha subunit |
| K17697 | DOCK4; dedicator of cytokinesis protein 4 |
| K07151 | STT3; dolichyl-diphosphooligosaccharide---protein glycosyltransferase |
| K25639 | SUPT16H, SPT16; FACT complex subunit SPT16 |
| K11592 | DICER1, DCR1; endoribonuclease Dicer |
| K03549 | kup; KUP system potassium uptake protein |
| K21435 | ANKRD6; ankyrin repeat domain-containing protein 6 |
| K04077 | groEL, HSPD1, HSP60, CPN60; chaperonin GroEL |
| K06158 | ABCF3; ATP-binding cassette, subfamily F, member 3 |

Table 4. Expanded protein/domain families in *Nitzschia* sp., annotated using Pfam.

| Pfam no. | Pfam annotation |
| --- | --- |
| PF09011 | HMG-box domain |
| PF00505 | HMG (high mobility group) box |
| PF01872 | RibD C-terminal domain |
| PF05462 | Slime mold cyclic AMP receptor |
| PF00083 | Sugar (and other) transporter |
| PF03134 | TB2/DP1, HVA22 family |
| PF05699 | hAT family C-terminal dimerisation region |
| PF02028 | BCCT, betaine/carnitine/choline family transporter |
| PF03016 | Exostosin family |
| PF01457 | Leishmanolysin |
| PF02892 | BED zinc finger |
| PF01822 | WSC domain |
| PF00001 | 7 transmembrane receptor (rhodopsin family) |
| PF06990 | Galactose-3-O-sulfotransferase |
| PF17783 | CvfB-like winged helix domain |
| PF10294 | Lysine methyltransferase |
| PF00917 | MATH domain |

Table 5. Expanded protein families in *Nitzschia* sp., annotated using KEGG.

| KEGG no. | KEGG annotation |
| --- | --- |
| K24193 | STP; MFS transporter, SP family, sugar:H+ symporter |
| K11752 | ribD; diaminohydroxyphosphoribosylaminopyrimidine deaminase / 5-amino-6-(5-phosphoribosylamino)uracil reductase |
| K06891 | clpS; ATP-dependent Clp protease adaptor protein ClpS |
| K09250 | CNBP; cellular nucleic acid-binding protein |
| K15711 | SMARCA3, HLTF; SWI/SNF-related matrix-associated actin-dependent regulator of chromatin subfamily A3 |
| K01338 | lon; ATP-dependent Lon protease |
| K03218 | rlmB; 23S rRNA (guanosine2251-2'-O)-methyltransferase |
| K14163 | EPRS; bifunctional glutamyl/prolyl-tRNA synthetase |
| K01076 | ABHD17; abhydrolase domain-containing protein 17 |
| K00600 | glyA, SHMT; glycine hydroxymethyltransferase |
| K04229 | OXTR; oxytocin receptor |
| K10802 | HMGB1; high mobility group protein B1 |
| K11295 | HMGB2; high mobility group protein B2 |
| K09272 | SSRP1, POB3; FACT complex subunit SSRP1/POB3 |
| K11296 | HMGB3; high mobility group protein B3 |
| K05751 | ABI2; abl interactor 2 |
| K13291 | TUT; terminal uridylyltransferase |
| K01870 | IARS, ileS; isoleucyl-tRNA synthetase |
| K04759 | feoB; ferrous iron transport protein B |
| K24637 | ZBED1; zinc finger BED domain-containing protein 1 (E3 SUMO-protein ligase ZBED1) |
| K02168 | betT, betS; choline/glycine/proline betaine transport protein |

Table 6. Contracted protein/domain families in *Nitzschia* sp., annotated using Pfam.

| Pfam no. | Pfam annotation |
| --- | --- |
| PF12738 | twin BRCT domain |
| PF00612 | IQ calmodulin-binding motif |
| PF00063 | Myosin head (motor domain) |
| PF07714 | Protein tyrosine and serine/threonine kinase |
| PF12974 | ABC transporter, phosphonate, periplasmic substrate-binding protein |
| PF00211 | Adenylate and Guanylate cyclase catalytic domain |
| PF00089 | Trypsin |
| PF00233 | 3'5'-cyclic nucleotide phosphodiesterase |
| PF00060 | Ligand-gated ion channel |
| PF17830 | STI1 domain |
| PF17917 | RNase H-like domain found in reverse transcriptase |
| PF00067 | Cytochrome P450 |
| PF03382 | Mycoplasma protein of unknown function, DUF285 |
| PF13306 | BspA type Leucine rich repeat region (6 copies) |
| PF00082 | Subtilase family |
| PF13191 | AAA ATPase domain |
| PF03842 | Silicon transporter |
| PF05787 | Bacterial protein of unknown function (DUF839) |
| PF13639 | Ring finger domain |
| PF00394 | Multicopper oxidase |
| PF07732 | Multicopper oxidase |
| PF07731 | Multicopper oxidase |
| PF05547 | Immune inhibitor A peptidase M6 |
| PF07700 | Haem-NO-binding |
| PF07701 | Heme NO binding associated |
| PF03600 | Citrate transporter |
| PF02080 | TrkA-C domain |
| PF00955 | HCO3- transporter family |
| PF13499 | EF-hand domain pair |
| PF02184 | HAT (Half-A-TPR) repeat |
| PF02214 | BTB/POZ domain |
| PF00168 | C2 domain |
| PF17921 | Integrase zinc binding domain |
| PF02493 | MORN repeat |
| PF01926 | 50S ribosome-binding GTPase |
| PF12678 | RING-H2 zinc finger domain |
| PF05050 | Methyltransferase FkbM domain |
| PF03031 | NLI interacting factor-like phosphatase |
| PF01753 | MYND finger |
| PF00504 | Chlorophyll A-B binding protein |
| PF01612 | 3'-5' exonuclease |
| PF13516 | Leucine Rich repeat |
| PF01554 | MatE |
| PF04928 | Poly(A) polymerase central domain |
| PF04926 | Poly(A) polymerase predicted RNA binding domain |
| PF01483 | Proprotein convertase P-domain |
| PF02690 | Na+/Pi-cotransporter |
| PF13976 | GAG-pre-integrase domain |
| PF05922 | Peptidase inhibitor I9 |
| PF00856 | SET domain |
| PF01205 | Uncharacterized protein family UPF0029 |
| PF13532 | 2OG-Fe(II) oxygenase superfamily |
| PF04488 | Glycosyltransferase sugar-binding region containing DXD motif |
| PF00009 | Elongation factor Tu GTP binding domain |
| PF13424 | Tetratricopeptide repeat |
| PF04749 | PLAC8 family |
| PF01412 | Putative GTPase activating protein for Arf |
| PF03457 | Helicase associated domain |
| PF01062 | Bestrophin, RFP-TM, chloride channel |
| PF11700 | Vacuole effluxer Atg22 like |
| PF00350 | Dynamin family |
| PF00512 | His Kinase A (phospho-acceptor) domain |
| PF04851 | Type III restriction enzyme, res subunit |
| PF00497 | Bacterial extracellular solute-binding proteins, family 3 |
| PF00003 | 7 transmembrane sweet-taste receptor of 3 GCPR |
| PF00651 | BTB/POZ domain |
| PF03024 | Folate receptor family |
| PF01593 | Flavin containing amine oxidoreductase |
| PF18031 | Ubiquitin carboxyl-terminal hydrolases |
| PF01088 | Ubiquitin carboxyl-terminal hydrolase, family 1 |
| PF01094 | Receptor family ligand binding region |
| PF01909 | Nucleotidyltransferase domain |
| PF18761 | Heliorhodopsin |
| PF00650 | CRAL/TRIO domain |
| PF05843 | Suppressor of forked protein (Suf) |
| PF05686 | Glycosyl transferase family 90 |
| PF04545 | Sigma-70, region 4 |
| PF04542 | Sigma-70 region 2 |
| PF04515 | Plasma-membrane choline transporter |
| PF02671 | Paired amphipathic helix repeat |
| PF06293 | Lipopolysaccharide kinase (Kdo/WaaP) family |
| PF00560 | Leucine Rich Repeat |
| PF00795 | Carbon-nitrogen hydrolase |
| PF07885 | Ion channel |
| PF13374 | Tetratricopeptide repeat |
| PF06468 | Spondin_N |
| PF00665 | Integrase core domain |
| PF07690 | Major Facilitator Superfamily |
| PF13637 | Ankyrin repeats (many copies) |
| PF13923 | Zinc finger, C3HC4 type (RING finger) |
| PF01661 | Macro domain |
| PF13176 | Tetratricopeptide repeat |
| PF00176 | SNF2-related domain |
| PF00271 | Helicase conserved C-terminal domain |
| PF13383 | Methyltransferase domain |
| PF00533 | BRCA1 C Terminus (BRCT) domain |
| PF13181 | Tetratricopeptide repeat |
| PF04886 | PT repeat |
| PF01079 | Hint module |
| PF02469 | Fasciclin domain |
| PF02825 | WWE domain |
| PF00077 | Retroviral aspartyl protease |
| PF01007 | Inward rectifier potassium channel transmembrane domain |
| PF09353 | Domain of unknown function (DUF1995) |
| PF13450 | NAD(P)-binding Rossmann-like domain |
| PF00097 | Zinc finger, C3HC4 type (RING finger) |
| PF13704 | Glycosyl transferase family 2 |
| PF00676 | Dehydrogenase E1 component |
| PF08007 | JmjC domain |
| PF17874 | MalT-like TPR region |
| PF09739 | Mini-chromosome maintenance replisome factor |
| PF02771 | Acyl-CoA dehydrogenase, N-terminal domain |
| PF00441 | Acyl-CoA dehydrogenase, C-terminal domain |
| PF17655 | Inward rectifier potassium channel C-terminal domain |
| PF10459 | Peptidase S46 |
| PF02492 | CobW/HypB/UreG, nucleotide-binding domain |
| PF07683 | Cobalamin synthesis protein cobW C-terminal domain |
| PF03924 | CHASE domain |
| PF02037 | SAP domain |
| PF02566 | OsmC-like protein |
| PF12861 | Anaphase-promoting complex subunit 11 RING-H2 finger |
| PF01148 | Cytidylyltransferase family |
| PF03479 | Plants and Prokaryotes Conserved (PCC) domain |
| PF12796 | Ankyrin repeats (3 copies) |
| PF00232 | Glycosyl hydrolase family 1 |
| PF13599 | Pentapeptide repeats (9 copies) |
| PF05830 | Nodulation protein Z (NodZ) |
| PF00652 | Ricin-type beta-trefoil lectin domain |
| PF08547 | Complex I intermediate-associated protein 30 (CIA30) |
| PF00106 | short chain dehydrogenase |
| PF03737 | Aldolase/RraA |
| PF13460 | NAD(P)H-binding |
| PF13833 | EF-hand domain pair |
| PF00378 | Enoyl-CoA hydratase/isomerase |
| PF13202 | EF hand |
| PF10184 | Uncharacterized conserved protein (DUF2358) |
| PF01764 | Lipase (class 3) |
| PF04434 | SWIM zinc finger |
| PF05347 | Complex 1 protein (LYR family) |

Table 7. Contracted protein families in *Nitzschia* sp., annotated using KEGG.

| KEGG no. | KEGG annotation |
| --- | --- |
| K12324 | ANPRB, NPR2; atrial natriuretic peptide receptor B |
| K12322 | GUCY2F; guanylate cyclase 2F |
| K04998 | KCNJ4, KIR2.3; potassium inwardly-rectifying channel subfamily J member 4 |
| K04996 | KCNJ2, KIR2.1; potassium inwardly-rectifying channel subfamily J member 2 |
| K05002 | KCNJ9, KIR3.3; potassium inwardly-rectifying channel subfamily J member 9 |
| K05000 | KCNJ6, KIR3.2; potassium inwardly-rectifying channel subfamily J member 6 |
| K00884 | NAGK, nagK; N-acetylglucosamine kinase |
| K21918 | KCTD8_12_16; BTB/POZ domain-containing protein KCTD8/12/16 |
| K21922 | KCTD21; BTB/POZ domain-containing protein KCTD21 |
| K21915 | KCTD3; BTB/POZ domain-containing protein KCTD3 |
| K21921 | KCTD18; BTB/POZ domain-containing protein KCTD18 |
| K04889 | KCNC3, KV3.3; potassium voltage-gated channel Shaw-related subfamily C member 3 |
| K21754 | KCTD1_15; BTB/POZ domain-containing protein KCTD1/15 |
| K19199 | SETD3; protein-histidine N-methyltransferase |
| K11430 | EZH2; [histone H3]-lysine27 N-trimethyltransferase EZH2 |
| K10357 | MYO5; myosin V |
| K10352 | MYH9s; myosin heavy chain 9/10/11/14 |
| K15701 | RNF130, GOLIATH; E3 ubiquitin-protein ligase RNF130 |
| K11982 | RNF115_126; E3 ubiquitin-protein ligase RNF115/126 |
| K10629 | RNF128, GRAIL; E3 ubiquitin-protein ligase RNF128 |
| K09091 | HEY; hairy and enhancer of split related with YRPW motif |
| K10450 | KLHL12, C3IP1; kelch-like protein 12 |
| K07093 | K07093; uncharacterized protein |
| K12319 | GUCY1B; guanylate cyclase soluble subunit beta |
| K26126 | SLF1; SMC5-SMC6 complex localization factor protein 1 |
| K11827 | AP2S1; AP-2 complex subunit sigma-1 |
| K23342 | FBN2_3; fibrillin 2/3 |
| K04550 | LRP1, CD91; low-density lipoprotein receptor-related protein 1 (alpha-2-macroglobulin receptor) |
| K20049 | LRP1B; low-density lipoprotein receptor-related protein 1B |
| K11990 | DHH; desert hedgehog |
| K19413 | KDM1B, AOF1, LSD2; lysine-specific histone demethylase 1B |
| K08672 | PCSK6; proprotein convertase subtilisin/kexin type 6 |
| K01359 | PCSK1; proprotein convertase subtilisin/kexin type 1 |
| K08673 | PCSK7; proprotein convertase subtilisin/kexin type 7 |
| K01341 | KEX2; kexin |
| K08654 | PCSK5; proprotein convertase subtilisin/kexin type 5 |
| K01349 | FURIN, PCSK3; furin |
| K03695 | clpB; ATP-dependent Clp protease ATP-binding subunit ClpB |
| K26544 | SEC14, SEC14L; phosphatidylinositol/phosphatidylcholine transfer protein |
| K12030 | TRIM62; tripartite motif-containing protein 62 |
| K13172 | SRRM2, SRM300; serine/arginine repetitive matrix protein 2 |
| K12486 | SMAP; stromal membrane-associated protein |
| K17848 | AGAP2; Arf-GAP with GTPase, ANK repeat and PH domain-containing protein 2 |
| K19360 | NPHP3; nephrocystin-3 |
| K09667 | OGT; protein O-GlcNAc transferase |
| K10407 | KLC; kinesin light chain |
| K09592 | EGLN, HPH; hypoxia-inducible factor prolyl hydroxylase |
| K01188 | E3.2.1.21; beta-glucosidase |
| K05207 | GRID2; glutamate receptor delta-2 subunit |
| K05208 | GRIN1; glutamate receptor ionotropic, NMDA 1 |
| K15732 | CTDP1, FCP1; RNA polymerase II subunit A C-terminal domain phosphatase |
| K13861 | SLC4A10, NCBE; solute carrier family 4 (sodium bicarbonate transporter), member 10 |
| K06573 | SLC4A1, AE1, CD233; solute carrier family 4 (anion exchanger), member 1 |
| K13855 | SLC4A2, AE2; solute carrier family 4 (anion exchanger), member 2 |
| K13856 | SLC4A3, AE3; solute carrier family 4 (anion exchanger), member 3 |
| K06689 | UBE2D, UBC4, UBC5; ubiquitin-conjugating enzyme E2 D |
| K07953 | SAR1; GTP-binding protein SAR1 |
| K03267 | ERF3, GSPT; peptide chain release factor subunit 3 |
| K16914 | RIOX1, NO66; protein-L-histidine (3S)-3-hydroxylase / [histone H3]-trimethyl-L-lysine4/36 demethylase |
| K12663 | ECH1; Delta3,5-Delta2,4-dienoyl-CoA isomerase |
| K14683 | SLC34A, NPT, nptA; solute carrier family 34 (sodium-dependent phosphate cotransporter) |
| K01312 | PRSS1_2_3; trypsin |
| K09628 | PRSS27; serine protease 27 |
| K05610 | UCHL5, UCH37; ubiquitin carboxyl-terminal hydrolase L5 |
| K12869 | CRN, CRNKL1, CLF1, SYF3; crooked neck |
| K13807 | PPEF, PPP7C; serine/threonine-protein phosphatase with EF-hands |
| K02553 | rraA, menG; regulator of ribonuclease activity A |
| K04445 | RPS6KA5, MSK1; ribosomal protein S6 kinase alpha-5 |
| K04373 | RPS6KA; ribosomal protein S6 kinase alpha-1/2/3/6 |
| K12323 | ANPRA, NPR1; atrial natriuretic peptide receptor A |
| K04615 | GABBR; gamma-aminobutyric acid type B receptor |
| K04613 | V2R; vomeronasal 2 receptor |
| K04611 | MXR; metabotropic X receptor |
| K06584 | ITGA8; integrin alpha 8 |
| K04924 | KCNK16, K2P16.1; potassium channel subfamily K member 16 |
| K05389 | KCNKF; potassium channel subfamily K, other eukaryote |
| K03327 | SLC47A, MATE, DTX; MATE family, multidrug and toxin extrusion protein |
| K12176 | COPS2, CSN2, TRIP15; COP9 signalosome complex subunit 2 |
| K08333 | PIK3R4, VPS15; phosphoinositide-3-kinase, regulatory subunit 4 |
| K06445 | fadE; acyl-CoA dehydrogenase |
| K02213 | CDC6; cell division control protein 6 |
| K01988 | A4GALT; lactosylceramide 4-alpha-galactosyltransferase |
| K00889 | PIP5K; 1-phosphatidylinositol-4-phosphate 5-kinase |
| K19755 | RSPH1; radial spoke head protein 1 |
| K13649 | FOLR; folate receptor |
| K25530 | IZUMO1R, FOLR4; sperm-egg fusion protein Juno |
| K20718 | ER; LRR receptor-like serine/threonine-protein kinase ERECTA |
| K08994 | yneE, BEST; ion channel-forming bestrophin family protein |
| K28063 | ESYT, TCB, SYT; extended synaptotagmin |
| K28058 | MCTP, FTIP; multiple C2 and transmembrane domain-containing protein |
| K19663 | PRKCG; classical protein kinase C gamma type |
| K19909 | SYT9; synaptotagmin-9 |
| K24473 | TENM, ODZ; teneurin |
| K24333 | MEGF6; multiple epidermal growth factor-like domains protein 6 |
| K00488 | CYP27A1; cholestanetriol 26-monooxygenase |
| K21953 | SHKBP1; SH3KBP1-binding protein 1 |
| K21917 | KCTD7_14; BTB/POZ domain-containing protein KCTD7/14 |
| K12321 | GUCY2D_E; guanylate cyclase 2D/E |
| K14376 | PAP; poly(A) polymerase |
| K01769 | E4.6.1.2; guanylate cyclase, other |

Table 8. Transposable element (TE) statistics on eight *Bacillariophyceae* diatoms. All statistics are expressed as percentages relative to the total genome size.

| Diatom species | all TE | LTR element | T1/Copia | Unclassified |
| --- | --- | --- | --- | --- |
| *Cylindrotheca_closterium* | 22.75 | 7.55 | 5.83 | 11.44 |
| *Fistulifera_solaris* | 6.69 | 1.05 | 1.04 | 4.04 |
| *Fragilariopsis_cylindrus* | 17.06 | 3.31 | 2.43 | 7.25 |
| *Nitzschia_inconspicua* | 24.36 | 3.38 | 2.25 | 13.07 |
| *Nitzschia sp.* | 27.25 | 5.5 | 4.73 | 14.75 |
| *Phaeodactylum_tricornutum* | 8.12 | 5.03 | 5.03 | 2.04 |
| *Pseudonitzschia_multistriata* | 25.45 | 2.79 | 1.5 | 16.25 |
| *Seminavis_robusta* | 26.5 | 7.34 | 3.63 | 14.73 |
